# Nebulized Fibrinolytic Agents Improve Pulmonary Fibrinolysis but Not Inflammation in Rat Models of Direct and Indirect Acute Lung Injury

**DOI:** 10.1371/journal.pone.0055262

**Published:** 2013-02-07

**Authors:** Jorrit J. Hofstra, Alexander D. Cornet, Paul J. Declerck, Barry Dixon, Hamid Aslami, Alexander P. J. Vlaar, Joris J. Roelofs, Tom van der Poll, Marcel Levi, Marcus J. Schultz

**Affiliations:** 1 Laboratory of Experimental Intensive Care and Anesthesiology, Academic Medical Centre, University of Amsterdam, Amsterdam, The Netherlands; 2 Department of Intensive Care Medicine, Academic Medical Centre, University of Amsterdam, Amsterdam, The Netherlands; 3 Department of Anesthesiology, Academic Medical Centre, University of Amsterdam, Amsterdam, The Netherlands; 4 Department of Medical Microbiology, Academic Medical Centre, University of Amsterdam, Amsterdam, The Netherlands; 5 Laboratory for Pharmaceutical Biology and Pharmacology, Faculty of Pharmaceutical Sciences, University of Leuven, Leuven, Belgium; 6 Department of Intensive Care Medicine, St. Vincent’s Hospital, Melbourne, Australia; 7 Department of Internal Medicine, Academic Medical Centre, University of Amsterdam, Amsterdam, The Netherlands; 8 Department of Pathology, Academic Medical Centre, University of Amsterdam, Amsterdam, The Netherlands; 9 Centre for Experimental and Molecular Medicine, Academic Medical Centre, University of Amsterdam, Amsterdam, The Netherlands; 10 HERMES Critical Care Group, Amsterdam, The Netherlands; The Ohio State University, United States of America

## Abstract

**Background:**

Critically ill patients frequently develop acute lung injury (ALI). Disturbed alveolar fibrin turnover, a characteristic feature of ALI, is the result of both activation of coagulation and inhibition of fibrinolysis. Nebulized fibrinolytic agents could exert lung–protective effects, via promotion of fibrinolysis as well as anti–inflammation.

**Methods:**

Rats were challenged intratracheally with *Pseudomonas aeruginosa*, resulting in pneumonia as a model for direct ALI, or received an intravenous bolus infusion of lipopolysaccharide, as a model for indirect ALI. Rats were randomized to nebulization of normal saline (placebo), recombinant tissue plasminogen activator (rtPA), or monoclonal antibodies against plasminogen activator inhibitor–type 1 (anti–PAI–1).

**Results:**

Nebulized rtPA or anti–PA1–1 enhanced the bronchoalveolar fibrinolytic system, as reflected by a significant reduction of PAI–1 activity levels in bronchoalveolar lavage fluid, and a consequent increase in plasminogen activator activity (PAA) levels to supranormal values. Both treatments also significantly affected systemic fibrinolysis as reflected by a significant increase in PAA levels in plasma to supranormal levels. Neither nebulized rtPA nor anti–PA1–1 affected pulmonary inflammation. Neither treatment affected bacterial clearance of *P. aeruginosa* from the lungs in case of pneumonia.

**Conclusions:**

Local treatment with rtPA or anti–PA1–1 affects pulmonary fibrinolysis but not inflammation in models of direct or indirect ALI in rats.

## Introduction

Acute lung injury (ALI) is a frequent complication of critical illness with high morbidity and mortality [1]. ALI may develop either from a direct pulmonary insult (e.g., pneumonia) or secondary to a systemic insult (e.g., sepsis). Local disturbances in coagulation and fibrinolysis are intrinsic to ALI [2], [3], and pulmonary coagulopathy is suggested to play a pivotal role in the pathogenesis of lung injury [4]. Indeed, nearly all patients with ALI demonstrate abnormalities in alveolar turnover of fibrin, varying from subtle changes in molecular markers of coagulation and fibrinolysis to more evident fibrin depositions in the smaller airways [5]. Reduced pulmonary fibrinolysis seems an important feature of pulmonary coagulopathy, independent of the origin of pulmonary inflammation. Reduced breakdown of fibrin depositions is, at least in part, the result of increased production of plasminogen activator inhibitor (PAI)–1, the main inhibitor of plasminogen activator in the lungs [6].

Theoretical considerations suggest that targeting pulmonary coagulation imbalance by enhancing fibrinolysis may benefit patients with ALI. Fibrin may not be merely an end–product of coagulation, but may also exaggerate or even initiate lung injury. Fibrin depositions activate neutrophils and fibroblasts, decrease alveolar fluid clearance (thereby inactivating surfactant and favoring alveolar collapse [7], [8]), increase pulmonary dead space and cause additional endothelial injury [9]. Fibrin, however, has also been found to be involved in regulating the inflammatory response that restores structure and function to injured tissues [10]. Monocytes and fibroblasts are able to bind to fibrin, increasing the inflammatory response by facilitating and enhancing cell migration, eventually leading to lung fibrosis [11], [12]. Reduced fibrinolysis, however, may also be seen as a beneficial response since it may help to contain inflammatory activity, or even infectious pathogens, to the site of injury.

Fibrinolytic agents are generally administered intravenously, resulting in systemic increase in fibrinolysis. Fibrinolytic therapy, consequently, is associated with sometimes life–threatening bleedings. Indeed, up to 7% of patients subjected to fibrinolytic therapy require blood transfusions, and up to 1% die because of bleedings [13]. Since coagulopathy in ALI is mainly restricted to the pulmonary compartment local administration through nebulization could increase local efficacy while minimizing the risk of bleeding.

The potential use of systemic infusion of recombinant tissue–type plasminogen activator (rtPA) has been explored previously in various models of direct and indirect ALI [14]–[18]. The potential use of anti–PA1–1 monoclonal antibodies [19] has never been tested in models of ALI, but anti–PA1–1 was found to be an effective pro–fibrinolytic agent in a rat model of intestinal ischemia reperfusion injury [17]. In the present study we investigated the effect of local administration of these two pro–fibrinolytic agents in two rat models of ALI: one model for direct ALI (*Pseudomonas aeruginosa* pneumonia) and one for direct ALI (systemic challenge with endotoxin). We hypothesized that nebulized pro–fibrinolytics increase breakdown of alveolar fibrin, and attenuate pulmonary inflammation.

## Materials and Methods

### Animals

The Institutional Animal Care and Use Committee of the Academic Medical Center approved all experiments. Animals were handled in accordance with the guidelines prescribed by international and national legislation on protection, care, and handling of laboratory animals. The study included 21 male Sprague–Dawley rats (200–250 g) (Harlan, The Hague, The Netherlands), subjected to pneumonia or endotoxemia. 10 healthy male Sprague-Dawley rats were used in various control groups.

### Induction of Pneumonia

Pneumonia was induced by intratracheal instillation of 10^8^ colony–forming units (CFU) of *P. aeruginosa* (PAO1, in a total volume of 250 µL of bacterial suspension), Bacteria were cultured and harvested as described previously [20].

### Endotoxemia–induced Lung Injury

Endotoxemia and subsequent lung injury was induced by intravenous bolus infusion of 7.5 mg/kg lipopolyssacharide (LPS) from *Escherichia coli* 0111:B4 (Sigma, St. Louis, MO, USA) through the penile vein under isoflurane (3%) anesthesia.

### Study Groups

Rats with *P. aeruginosa* pneumonia or endotoxemia–induced lung injury were randomized to nebulization of placebo (normal saline) (N = 7), treatment with clinical grade rtPA (N = 7) (Tenecteplase, Boehringer Ingelheim, Ingelheim, Germany) or anti–PA1–1 (N = 7) (anti–PAI1). Anti–PA1–1 monoclonal antibody (MA)–33H1F7 was produced as described previously [19]. Endotoxin levels for both products was <1 EU/mg. Unchallenged were treated with rtPA (N = 3) or –PA1–1 (N = 3) to evaluate the effect of nebulized medication alone on lung inflammation and cytokine levels. Unchallenged untreated rats served as controls (N = 4).

All agents were administered in a volume of 0.7 mL via nebulization 30 minutes before and at 6 and 12 hours after induction of pneumonia or endotoxemia. Total dosage of each agent was based using data from previous studies [14], [16], [17] and pilot studies with endotoxemia–induced lung injury while taking into account the efficacy of a nose–only exposure system and the possibility of each agent to dissolve to an acceptable volume for nebulization.

### Nebulization

For local administration of saline or study medication we used an exposure system, which allowed direct exposure of nebulized agents to the noses of rats with LPS–induced lung injury or pneumonia as described before [21]. This system includes a concentric manifold connected to the necks of bottle–like restraint tubes (CHT 249 restraint tube, CH technologies Inc., Westwood, New Jersey) in which the animals were confined with their noses adjacent to the bottle–necks. The bottles are readily removable and the device can be disassembled for cleaning, by removing the bottles and removing the manifold. The inhalation chamber is suitable to accommodate several rats at once. The aerosol atmosphere was generated using the Aeroneb Pro Nebulizer (Aerogen Ltd., Gallway, Ireland), a device with high efficiency of delivering aerosol to the lung. The aerosols were directed to the inhalation chamber by a constant oxygen flow (2 L/min).

The animals were accommodated to restraint tubes at several occasions in the week before the experiments. During the experiment spontaneously breathing rats were simultaneously exposed for 10 minutes to the designated aerosol atmosphere. The volume of drug solution left in the Aeroneb Pro Nebulizer, when the nebulization had ceased, was negligible.

### Blood and Tissue Sampling

At 16 hours after induction of pneumonia or endotoxemia, rats were sacrificed with an intramuscular injection of ketamine 45 mg/kg (Eurovet, Bladel, The Netherlands) and medetomidine 0.25 mg/kg (Novartis, Arnhem, The Netherlands). Blood was collected from the *vena cava inferior* in citrated (0.109 M) vacutainer tubes. The right lung was ligated, and the left lung was lavaged 3 times with 2 mL ice–cold normal saline. Right lungs were weighed and homogenized in 4 volumes (i.e., 4× lung weight [mg] in µL) of sterile saline using a tissue homogenizer (Biospec Products, Bartlesville, OK).

Total cell numbers in each lavage sample were determined with an automated cell counter (Z2 Coulter Pariticle Counter, Beckman Coulter Corporation, Hialeah, FL). Neutrophil counts in lavage fluids were performed on Giemsa–stained cytospin preparations.

Plasma and cell–free supernatants from lung lavage fluids were used for measuring coagulation. Lung homogenates were diluted 1∶1 in lysis buffer (150 nmol/L NaCl; 15 mmol/L Tris; 1 mmol/L MgCl_2_–H_2_O; 1 mmol/L CaCl_2_; 1% Triton X–100; and 100 µg/ml pepstatin A, leupeptin, and aprotinin) and used for cytokine and chemokine measurements.

### Assays

Thrombin–antithrombin complexes (TATc; Behring, Marburg, Germany) and fibrin degradation products (FDP; Diagnostica Stago, Asnières–sur–Seine, France) were measured in lung lavage fluid by means of ELISA. Antithrombin (AT), plasminogen activator activity (PAA), and plasminogen activator inhibitor (PAI)–1 activity were measured by an automated amidolytic assays [16]. Levels of tumor necrosis factor–α (TNF, R&D Systems, Abingdon, United Kingdom), interleukin (IL)–6 (R&D Systems, Abingdon, United Kingdom) and cytokine–induced neutrophil chemoattractant (CINC)–3 (R&D Systems, Abingdon, United Kingdom) and myeloperoxidase (MPO, HyCult biotechnology b.v., Uden, The Netherlands) were measured in lungs by means of ELISA.

### Histopathology

Lung sections, 4 µm in thickness, were stained with hematoxylin and eosin and analyzed and scored by two investigators who were blinded for group identity. To score lung inflammation and damage, the entire lung surface was analyzed with respect to the following variables: interstitial inflammation, endothelialitis/vasculitis, bronchitis, edema, pleuritis, and thrombus formation, as described previously [16]. The total histopathology score was expressed as the sum of the scores for all variables (on a scale of 0 to 4; 0, absent; 1, mild; 2, moderate; 3, severe; 4, very severe). The total lung injury score was calculated as sum of the scores for each category.

### Statistical Analyses

Comparisons between the experimental rat groups and saline–treated placebo rat group were performed using one–way analysis of variance (ANOVA) or Kruskal–Wallis test, followed by post–hoc Dunnett’s or Dunn’s tests, depending on data distribution. A *P*–value<0.05 was considered statistically significant. Statistical analyses were performed with SPSS 16.0 (SPSS, Chicago, IL, USA) and Prism 4.0 (GraphPad Software, San Diego, CA, USA).

## Results

### Clinical Signs after Challenge

Typical clinical symptoms of illness (pilo–erection, decreased activity, arched back, decreased food and water intake and increased respiratory rate) occurred shortly after intratracheal challenge with bacteria or intravenous bolus infusion of LPS. The animals averagely lost 8–10% of their bodyweight over the course of 16 hrs mainly due to dehydration. Rats sacrificed 16 hours after induction of pneumonia or endotoxemia had evident diffuse bilateral macroscopic lung abnormalities.

### Pulmonary Fibrinolysis and Coagulation

**Figure 1:**
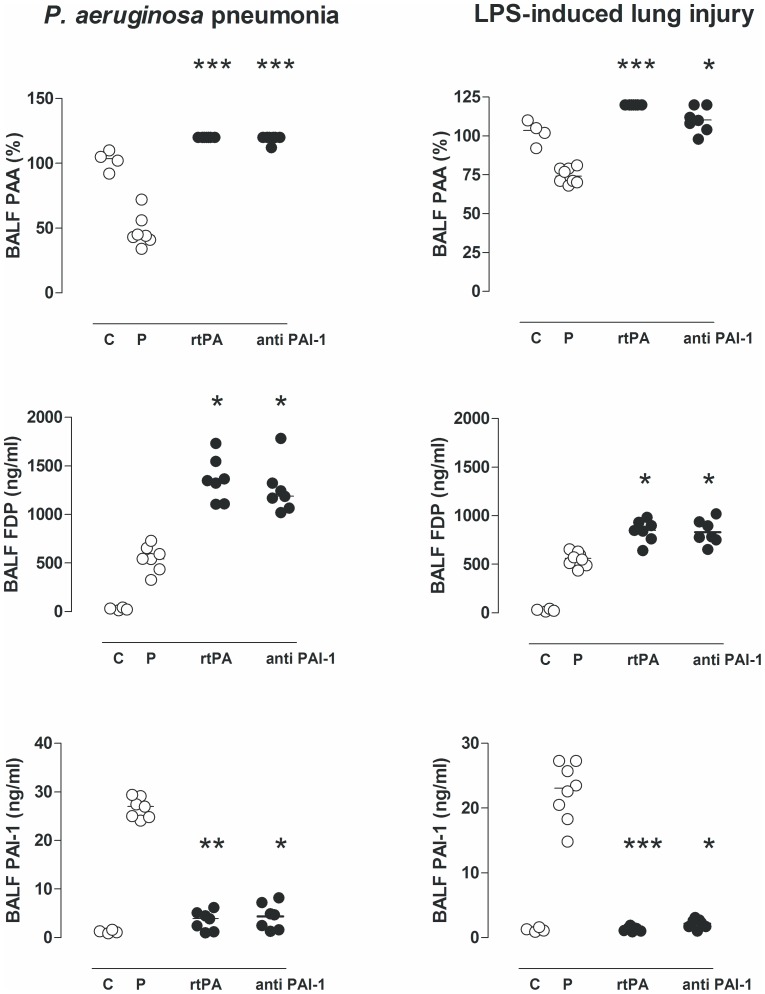
The effects of recombinant tissue type plasminogen activator (rtPA), anti–PA1–1 monoclonal antibodies (anti–PAI–1), or placebo (normal saline (P)) on levels of plasminogen activator activity (PAA), fibrin degradation products (FDP) and plasminogen activator inhibitor (PAI–1) in lavage fluid, 16 hours after intratracheal bacterial challenge (*Pseudomonas aeruginosa*, PAO1, 10^8^ colony forming units (CFU)) or 16 hours after intravenous injection of lipopolysaccharide (LPS, *Escherichia coli* O111:B4). The horizontal line within the graph represents the median. *, *p*<0.05 vs. saline; **, *p*<0.01 vs. saline; ***, *p*<0.001 vs. saline.

**Figure 2:**
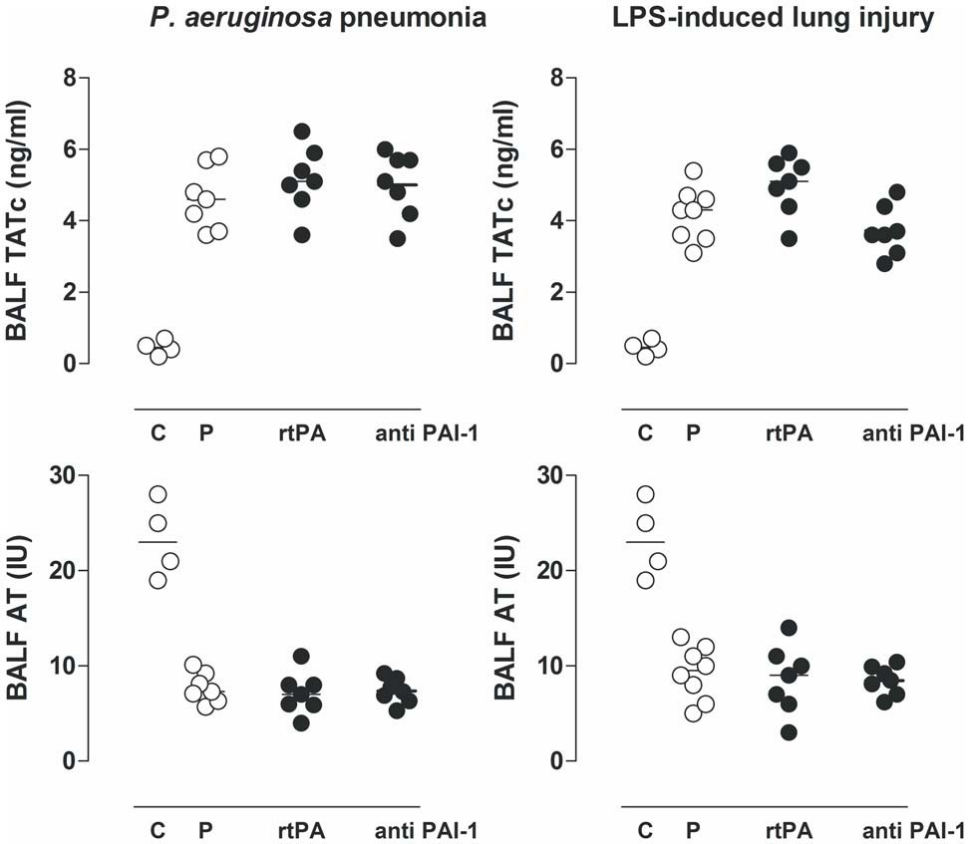
The effects of recombinant tissue type plasminogen activator (rtPA), anti–PA1–1 monoclonal antibodies (anti–PAI–1), or placebo (normal saline (P)) on levels of thrombin antithrombin complexes (TATc) and antithrombin (AT) in lavage fluid, 16 hours after intratracheal bacterial challenge (*Pseudomonas aeruginosa*, PAO1, 10^8^ colony forming units (CFU)) or 16 h after intravenous injection of lipopolysaccharide (LPS, *Escherichia coli* O111:B4). The horizontal line within the graph represents the median. *, *p*<0.05 vs. saline; **, *p*<0.01 vs. saline; ***, *p*<0.001 vs. saline.

Both *P. aeruginosa* pneumonia and endotoxemia–induced lung injury in rats was associated with pulmonary activation of coagulation and inhibition of fibrinolytic activity, reflected in increased levels of TATc, FDP and PAI–1 activity, and reduced levels of AT and PAA compared with uninfected controls. Nebulization of rtPA increased PAA to levels exceeding assay maximum in all rats in both models increasing fibrin degradation and abolishing PAI–1 activity. Nebulization of both rtPA and anti–PA1–1 eliminated (nearly) all free active PAI–1 resulting in significantly reduced pulmonary PAI–1 activity levels in both *P. aeruginosa* pneumonia and endotoxemia–induced lung injury. Increased fibrinolytic activity was reflected by a significant increase in FDP levels in both *P. aeruginosa* pneumonia (rtPA p<0.05 versus saline; and anti–rat PAI1 p<0.05 versus saline) and endotoxemia–induced lung injury (rtPA p<0.05 versus saline; and anti–rat PAI1 p<0.05 versus saline). Neither nebulized rtPA nor anti–rat PAI1 affected pulmonary levels of TATc and AT (figures 1 and 2).

### Systemic Coagulation and Fibrinolysis

**Figure 3:**
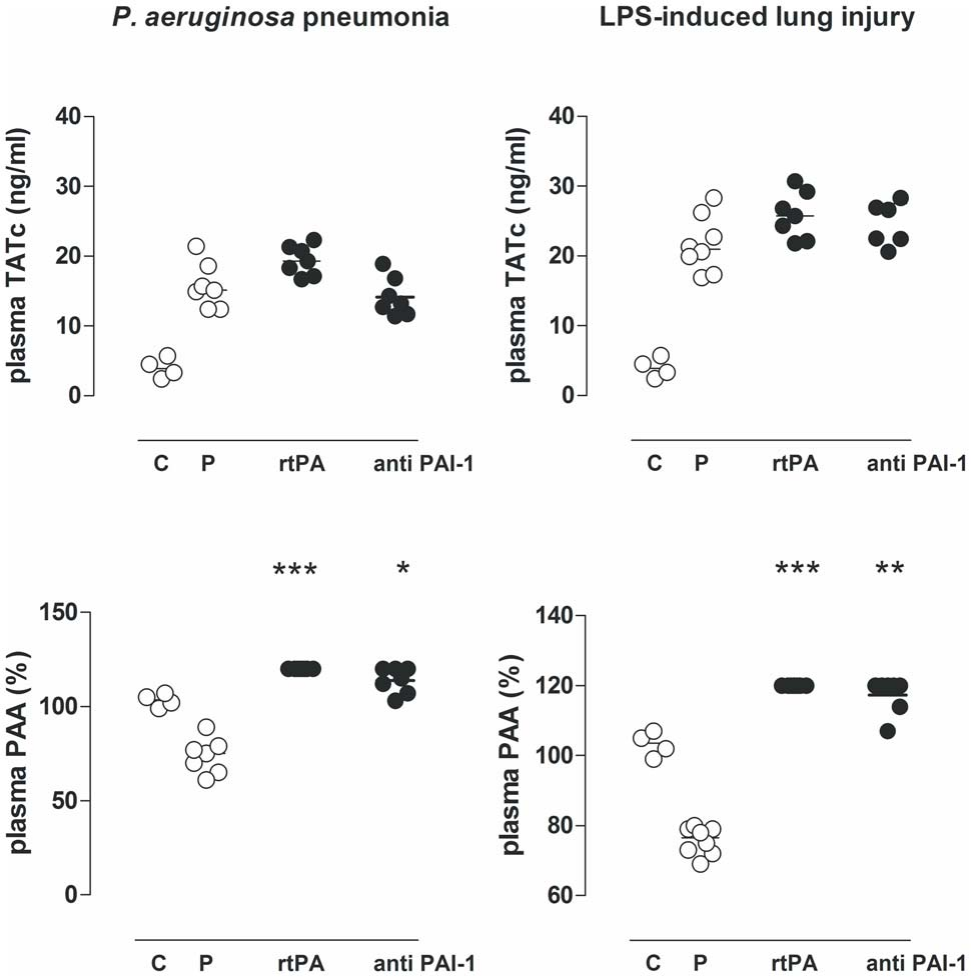
The effects of recombinant tissue type plasminogen activator (rtPA), anti–PA1–1 monoclonal antibodies (anti–PAI–1), or placebo (normal saline (P)) on levels of thrombin–antithrombin complexes (TATc) and plasminogen activator activity (PAA) in plasma, 16 hours after intratracheal bacterial challenge (*Pseudomonas aeruginosa*, PAO1, 10^8^ colony forming units (CFU)) or 16 hours after intravenous injection of lipopolysaccharide (LPS, *Escherichia coli* O111:B4). The horizontal line within the graph represents the median. *, *p*<0.05 vs. saline; **, *p*<0.01 vs. saline; ***, *p*<0.001 vs. saline.

Compared to healthy controls, challenge with intratracheal *P. aeruginosa* or intravenous LPS reduced plasma PAA and increased plasma levels of TATc. In rats with *P. aeruginosa* pneumonia both treatments greatly increased PAA. Similar effects were seen in endotoxemia–induced lung injury (figure 3).

### Bacterial Clearance from Lungs

Neither nebulized rtPA nor anti–PA1–1 affected bacterial clearance of *P. aeruginosa* from the lungs of rats with pneumonia. None of the animals developed bacteremia (data not shown).

### Inflammatory Response

**Figure 4:**
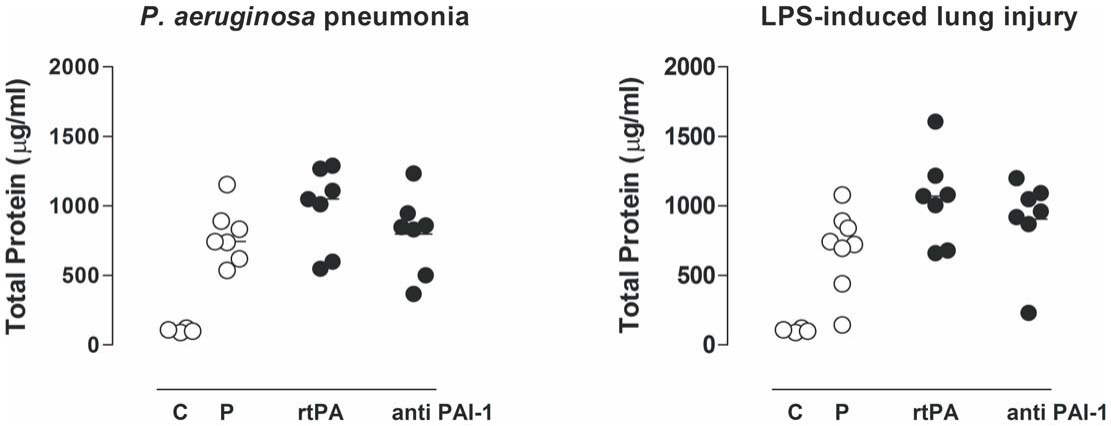
The effects of recombinant tissue type plasminogen activator (rtPA), anti–PA1–1 monoclonal antibodies (anti–PAI–1), or placebo (normal saline (P)) on total protein levels in lavage fluid, 16 hours after intratracheal bacterial challenge (*Pseudomonas aeruginosa*, PAO1, 10^8^ colony forming units (CFU)) or 16 h after intravenous injection of lipopolysaccharide (LPS, *Escherichia coli* O111:B4). The horizontal line within the graph represents the median.

**Figure 5:**
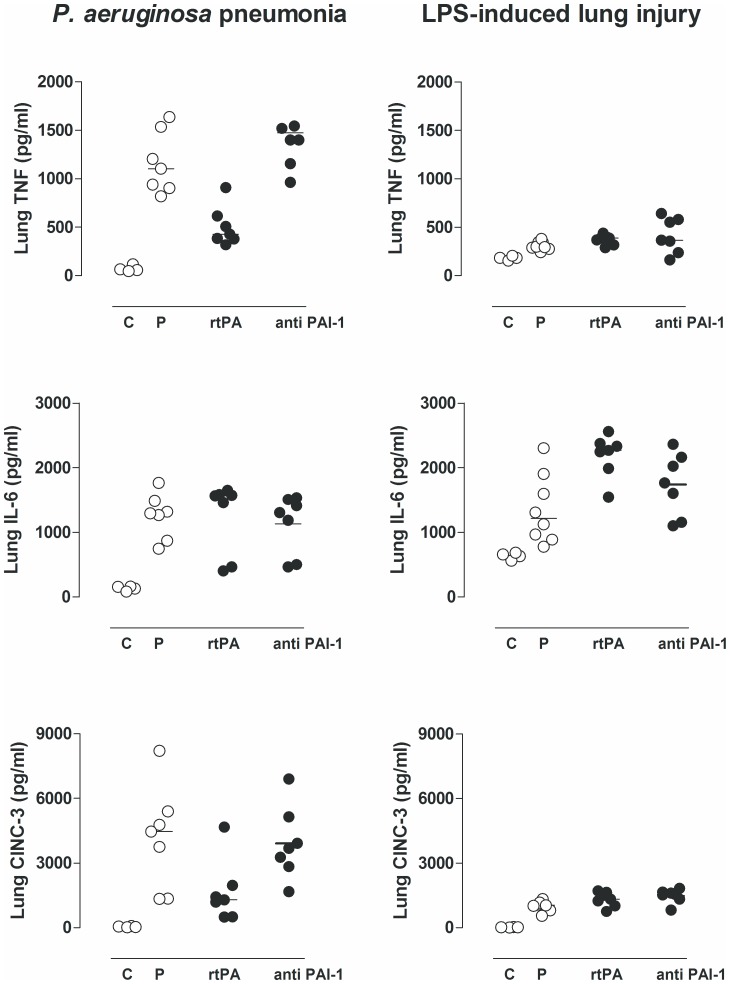
The effects of recombinant tissue type plasminogen activator (rtPA), anti–PA1–1 monoclonal antibodies (anti–PAI–1), or placebo (normal saline (P)) on levels of tumor necrosis factor α (TNF), interleukin (IL)–6, and cytokine–induced neutrophil chemoattractant (CINC)–3 in lung homogenates, 16 hours after intratracheal bacterial challenge (*Pseudomonas aeruginosa*, PAO1, 10^8^ colony forming units (CFU)) or 16 h after intravenous injection of lipopolysaccharide (LPS, *Escherichia coli* O111:B4). The horizontal line within the graph represents the median.

There was an evident increase in total cell number in the lungs during *P. aeruginosa* pneumonia and endotoxemia–induced lung injury, which was mostly contributed to neutrophil influx (table 1). The relative or absolute number of neutrophils in lavage fluids was not affected by any treatment (table 1), nor was the total protein concentration (figure 4). MPO was not altered (table 1) by any treatment in either model. There were no differences in pulmonary levels of TNF, IL–6, and CINC–3 during *P. aeruginosa* pneumonia or endotoxemia–induced lung injury (figure 5). Nebulization in healthy controls did not affect pulmonary inflammation as compared to untreated rats (data not shown).

**Table 1:** Total cell and neutrophil counts and myeloperoxidase (MPO) in bronchoalveolar lavage fluid of rats.

	Total cells	Neutrophils	MPO (µg/mL)
Controls	30 (14–51)	0 (0–0)	32 (21–51)
***Pseudomonas aeruginosa*** ** pneumonia (t = 16 h)**			
normal saline	203 (175–231)	204 (162–217)	551 (401–598)
rtPA	240 (170–269)	190 (126–207)	624 (599–644)
anti–PAI–1	283 (247–282)	256 (228–288)	663 (504–726)
**LPS–induced Lung Injury (t = 16 h)**			
normal saline	108 (74–141)	35 (24–51)	353 (278–404)
rtPA	140 (131–162)	29 (16–33)	318 (230–369)
anti–PAI–1	125 (113–148)	13 (12–25)	283 (258–310)

Cell counts 16 h after intratracheal instillation of *P. aeruginosa or* injection with lipopolysaccharide (*Escherischia coli* O111:B4). Controls are healthy rats (n = 4). Data are expressed as median (interquartile range)×10^4^ per milliliter of bronchoalveolar lavage fluid.

### Histopathology

**Figure 6:**
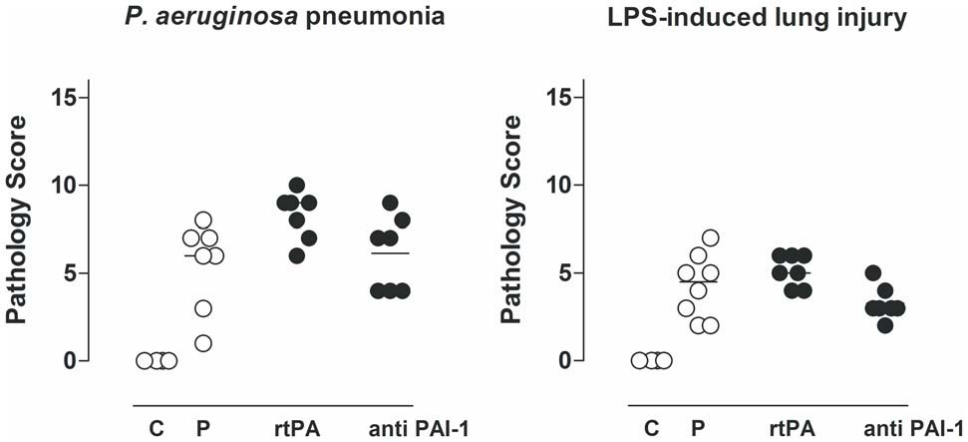
Histopathology scores of lung tissue from rats treated with recombinant tissue type plasminogen activator (rtPA), anti–PA1–1 monoclonal antibodies (anti–PAI–1), or placebo (normal saline (P)) 16 hrs after intratracheal bacterial challenge (*Pseudomonas aeruginosa*, PAO1, 10^8^ colony forming units (CFU)) or 16 h after intravenous injection of lipopolysaccharide (LPS, *Escherichia coli* O111:B4). The horizontal line within the graph represents the median.

**Figure 7:**
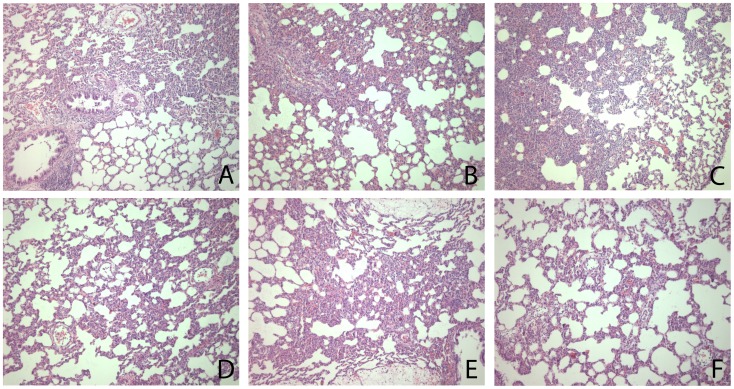
Histopathological changes in lungs of rats treated with recombinant tissue type plasminogen activator (rtPA), anti–PA1–1 monoclonal antibodies (anti–PAI–1), or placebo (normal saline (P)) 16 hrs after intratracheal bacterial challenge (*Pseudomonas aeruginosa*, PAO1, 10^8^ colony forming units (CFU)) or 16 h after intravenous injection of lipopolysaccharide (LPS, *Escherichia coli* O111:B4). Shown are representative hematoxylin and eosin–stained photomicrographs of lung tissue from rats treated with *Pseudomonas aeruginosa* + saline (A), *Pseudomonas aeruginosa* + rtPA (B), *Pseudomonas aeruginosa* + anti–PAI–1 (C), LPS + saline (D), LPS+rtPA (E), LPS + anti–PAI–1 (F), at 16 hrs after challenge.

At 16 hrs after inoculation with *P. aeruginosa*, histopathology of the lungs showed diffuse inflammatory infiltrates consisting chiefly of neutrophils. Interstitial inflammation, endothelialitis, bronchitis and edema were present to a variable extent. There were no differences in lung histopathology scores in any treatment group in either model (figures 6 and 7).

## Discussion

In this study we demonstrate that nebulization of rtPA or anti–PA1–1 considerably enhanced the bronchoalveolar fibrinolytic system in two models of ALI. Both nebulized rtPA and anti–PA1–1 treatment affected systemic fibrinolysis as well. However, neither rtPA nor anti–PA1–1 affected pulmonary inflammation or bacterial clearance from the respiratory tract.

These findings are in line with previous studies in rats investigating the effects of systemically administered rtPA in various models of acute lung injury [14], [16], [22]. In those studies rtPA did not affect pulmonary inflammation, or bacterial clearance form lungs in case of pneumonia. The results of the present study also seem in line with a study in a porcine model of trauma–induced ALI in which it was demonstrated that intravenous administration of tPA or uPA prevented hypoxemia and improved survival [23]. In addition, in a rat model of intratracheal IL–1–induced ALI, intraperitoneal administration of tPA decreased lung capillary leakage and attenuated neutrophil activation in the lungs [24] and tPA knockout mice have been shown to be protected from ischemia reperfusion lung injury [25]. Further in mice pulmonary transgenic tPA overexpression improved host defense during Klebsiella pneumonia [26]. Data from investigations on lung injury in PAI–1 deficient animals are inconclusive. Although PAI–1 knockout mice were protected from lung fibrosis after bleomycin induced lung injury [27], PAI–1 knockout mice were not protected against acid induced ALI [28]. Further PAI–1 knockout mice displayed worsened lung injury after pulmonary challenge with Klebsiella bacteria [29]. In our study we observed neither improvement nor worsening of pulmonary inflammation and lung injury.

Coagulation and inflammation are cardinal host defense mechanisms, mutually dependent in mounting an adequate immune response against potentially injurious challenges [30]. Interference with the procoagulant response may impede the primary host defense mechanisms as formation of thrombi is likely to be crucial in limiting hematogenic spread of bacteria in case of an infection. Although hematogenic dissemination of infection is suggested as a potential drawback of anticoagulant therapy in pneumonia [31], [32], none of the animals in our study developed bacteremia. It should be noted, though, that the set–up of our experiment may not have been optimal for detecting differences in bacterial spread.

The effects of both pro–fibrinolytic strategies on systemic fibrinolysis suggest that nebulized agents are leaking from the alveolar compartment into the circulation. Although it seems plausible, it is unclear whether this increase of systemic plasminogen activator activity results in a higher risk of bleeding, since our experiments were not designed to investigate these effects. So far, clinical studies testing the efficacy of locally applied anti–coagulant agents are sparse. In a single–center phase 1 trial of mechanically ventilated patients with ALI, nebulized heparin resulted in minimally prolonged systemic clotting times. Due to the design, no conclusions could be drawn regarding clinical effects, however no adverse events were reported, and nebulization was deemed feasible and safe in patients with ALI [33]. In a randomized controlled trial of patients at risk for ALI by the same investigators, the occurrence of blood–stained sputum was similar in the heparin and placebo groups. Blood product usage was also similar for the two groups, and no patients had blood loss or transfusion requirements attributable to the study medication [34]. In our study, possibly a lower dose would have achieved similar effects on pulmonary fibrinolysis while preventing systemic fibrinolysis to be affected. On the other hand, higher dosages may be required to achieve anti–inflammatory effects.

Fibrin is considered a key player in host defense. Fibrin is involved in regulating the inflammatory response that restores structure and function to injured tissues [10] Fibrin allows inflammatory cells as well as alveolar epithelial cells to migrate to the site of infection and is essential in the regulation and facilitation of pulmonary repair. Enhancing fibrinolysis may impair the beneficial effects and therefore potential beneficial effects on pulmonary inflammation may be overshadowed.

There are several limitations to our study. First, results in rat models for ALI used in these experiments may not be easily translated into studies of complex and heterogeneous patient populations. We have used a pre–treatment model, useful when exploring novel approaches and mechanisms. However, post–treatment models more closely resemble the clinical situation. Furthermore, in the *P. aeruginosa* pneumonia model no antibiotics were administered, which is different from any clinical scenario. Another disadvantage of our models is that they rely on a single high dose administration of LPS or live bacteria to healthy animals where ALI is usually associated with pre–existent risk factor and develops over a longer period of time. The chosen dosages for each anticoagulant agent were determined based on data from previous studies and pilot studies [14], [16], [17], combined with the efficacy of our nose–only exposure system, and the possibility of each agent to dissolve to an acceptable volume for nebulization. For both agents this approach led to a significant enhancement of pulmonary fibrinolysis, however as stated above a lower dose could possibly prevent systemic effects of treatment while an even higher dose may have been necessary to affect inflammation. Moreover, aerosol characteristics of the drugs studied probably could have been different. Frequency of administration of agents may be another limitation of the present study, as more frequent or continuous administration may have been more effective. Finally, bacteremia was not observed, but the observational period was only 16 hours and this model was not designed to evaluate its occurrence.

Another important limitation is that we did not investigate functional endpoints like arterial blood gas analysis or other gas exchange or pulmonary function parameters. Despite the absence of effects on inflammation it is possible that enhanced fibrinolysis improves pulmonary function in these rats.

In conclusion, pulmonary coagulopathy with ALI may allow for local interventions with pro–fibrinolytic agents. While nebulized rtPA and anti–PA1–1 affect pulmonary fibrinolysis, both strategies however did not affect pulmonary inflammation. Notably, both treatments affected systemic fibrinolysis.

## References

[1] Bernard GR, Artigas A, Brigham KL, Carlet J, Falke K, et al (1994) The American-European Consensus Conference on ARDS. Definitions, mechanisms, relevant outcomes, and clinical trial coordination. Am J Respir Crit Care Med 149: 818–824. 7509706 10.1164/ajrccm.149.3.7509706

[2] Dahlem P, Bos AP, Haitsma JJ, Schultz MJ, Wolthuis EK, et al (2006) Mechanical ventilation affects alveolar fibrinolysis in LPS-induced lung injury. The European respiratory journal : official journal of the European Society for Clinical Respiratory Physiology 28: 992–998. 10.1183/09031936.06.00133104 16837499

[3] Schultz MJ, Haitsma JJ, Zhang H, Slutsky AS (2006) Pulmonary coagulopathy as a new target in therapeutic studies of acute lung injury or pneumonia–a review. Critical care medicine 34: 871–877. 16521285

[4] Welty-Wolf KE, Carraway MS, Ortel TL, Piantadosi CA (2002) Coagulation and inflammation in acute lung injury. Thromb Haemost 88: 17–25. 12152667

[5] Gunther A, Mosavi P, Heinemann S, Ruppert C, Muth H, et al (2000) Alveolar fibrin formation caused by enhanced procoagulant and depressed fibrinolytic capacities in severe pneumonia. Comparison with the acute respiratory distress syndrome. American journal of respiratory and critical care medicine 161: 454–462. 10673185 10.1164/ajrccm.161.2.9712038

[6] Sawdey MS, Loskutoff DJ (1991) Regulation of murine type 1 plasminogen activator inhibitor gene expression in vivo. Tissue specificity and induction by lipopolysaccharide, tumor necrosis factor-alpha, and transforming growth factor-beta. The Journal of clinical investigation 88: 1346–1353. 1918385 10.1172/JCI115440 PMC295605

[7] Seeger W, Stohr G, Wolf HR, Neuhof H (1985) Alteration of surfactant function due to protein leakage: special interaction with fibrin monomer. J Appl Physiol 58: 326–338. 3838543 10.1152/jappl.1985.58.2.326

[8] Seeger W, Gunther A, Thede C (1992) Differential sensitivity to fibrinogen inhibition of SP-C- vs. SP-B-based surfactants. Am J Physiol 262: L286–L291. 1550251 10.1152/ajplung.1992.262.3.L286

[9] Ware LB (2006) Pathophysiology of acute lung injury and the acute respiratory distress syndrome. Semin Respir Crit Care Med 27: 337–349. 10.1055/s-2006-948288 [doi]. 10.1055/s-2006-948288 16909368

[10] Drew AF, Liu H, Davidson JM, Daugherty CC, Degen JL (2001) Wound-healing defects in mice lacking fibrinogen. Blood 97: 3691–3698. 11389004 10.1182/blood.v97.12.3691

[11] Idell S, Koenig KB, Fair DS, Martin TR, McLarty J, et al (1991) Serial abnormalities of fibrin turnover in evolving adult respiratory distress syndrome. Am J Physiol 261: L240–L248. 1928357 10.1152/ajplung.1991.261.4.L240

[12] Idell S, James KK, Levin EG, Schwartz BS, Manchanda N, et al (1989) Local abnormalities in coagulation and fibrinolytic pathways predispose to alveolar fibrin deposition in the adult respiratory distress syndrome. J Clin Invest 84: 695–705. 2788176 10.1172/JCI114217 PMC548934

[13] (1997) A comparison of reteplase with alteplase for acute myocardial infarction. The Global Use of Strategies to Open Occluded Coronary Arteries (GUSTO III) Investigators. N Engl J Med 337: 1118–1123. 10.1056/NEJM199710163371603 [doi]. 10.1056/NEJM199710163371603 9340503

[14] Choi G, Vlaar APJ, Schouten M, Van’t Veer C, Van der Poll T, et al (2007) Natural anticoagulants limit lipopolysaccharide-induced pulmonary coagulation but not inflammation. The European respiratory journal : official journal of the European Society for Clinical Respiratory Physiology 30: 423–428. 10.1183/09031936.00165606 17537762

[15] Choi G, Hofstra JJ, Roelofs JJ, Rijneveld AW, Bresser P, et al.. (2008) Antithrombin inhibits bronchoalveolar activation of coagulation and limits lung injury during Streptococcus pneumoniae pneumonia in rats. Crit Care Med 36: 204–210. 10.1097/01.CCM.0000292012.87482.F4 [doi]. 10.1097/01.CCM.0000292012.87482.F4 18090375

[16] Choi G, Hofstra JJ, Roelofs JJ, Florquin S, Bresser P, et al (2007) Recombinant human activated protein C inhibits local and systemic activation of coagulation without influencing inflammation during Pseudomonas aeruginosa pneumonia in rats. Critical care medicine 35: 1362–1368. 17414732 10.1097/01.CCM.0000261888.32654.6D

[17] Schoots IG, Levi M, van Vliet AnK, Declerck PJ, Maas AM, et al (2004) Enhancement of endogenous fibrinolysis does not reduce local fibrin deposition, but modulates inflammation upon intestinal ischemia and reperfusion. Thrombosis and haemostasis 91: 497–505. 14983225 10.1160/TH03-08-0529

[18] Stringer KA, Hybertson BM, Cho OJ, Cohen Z, Repine JE (1998) Tissue plasminogen activator (tPA) inhibits interleukin-1 induced acute lung leak. Free radical biology & medicine 25: 184–188. 9667494 10.1016/s0891-5849(98)00047-1

[19] Debrock S, Declerck PJ (1997) Neutralization of plasminogen activator inhibitor-1 inhibitory properties: identification of two different mechanisms. Biochimica et biophysica acta 1337: 257–266. 9048903 10.1016/s0167-4838(96)00173-2

[20] Schultz MJ, Rijneveld AW, Florquin S, Edwards CK, Dinarello CA, et al (2002) Role of interleukin-1 in the pulmonary immune response during Pseudomonas aeruginosa pneumonia. American journal of physiology Lung cellular and molecular physiology 282: L285–L290. 11792633 10.1152/ajplung.00461.2000

[21] Hofstra JJ, Vlaar AP, Cornet AD, Dixon B, Roelofs JJ, et al.. (2010) Nebulized anticoagulants limit pulmonary coagulopathy, but not inflammation, in a model of experimental lung injury. J Aerosol Med Pulm Drug Deliv 23: 105–111. 10.1089/jamp.2009.0779 [doi]. 10.1089/jamp.2009.0779 20073557

[22] Choi G, Hofstra JJH, Roelofs JJTH, Rijneveld AW, Bresser P, et al (2008) Antithrombin inhibits bronchoalveolar activation of coagulation and limits lung injury during Streptococcus pneumoniae pneumonia in rats. Crit Care Med 36: 204–210. 18090375 10.1097/01.CCM.0000292012.87482.F4

[23] Hardaway RM, Williams CH, Marvasti M, Farias M, Tseng A, et al (1990) Prevention of adult respiratory distress syndrome with plasminogen activator in pigs. Crit Care Med 18: 1413–1418. 2123144 10.1097/00003246-199012000-00021

[24] Stringer KA, Hybertson BM, Cho OJ, Cohen Z, Repine JE (1998) Tissue plasminogen activator (tPA) inhibits interleukin-1 induced acute lung leak. Free Radic Biol Med 25: 184–188. 9667494 10.1016/s0891-5849(98)00047-1

[25] Zhao Y, Sharma AK, Lapar DJ, Kron IL, Ailawadi G, et al.. (2011) Depletion of tissue plasminogen activator attenuates lung ischemia-reperfusion injury via inhibition of neutrophil extravasation. Am J Physiol Lung Cell Mol Physiol 300: L718–L729. ajplung.00227.2010 [pii];10.1152/ajplung.00227.2010 [doi]. 10.1152/ajplung.00227.2010 PMC3094027 21378024

[26] Renckens R, Roelofs JJ, Stegenga ME, Florquin S, Levi M, et al.. (2008) Transgenic tissue-type plasminogen activator expression improves host defense during Klebsiella pneumonia. J Thromb Haemost 6: 660–668. JTH2892 [pii];10.1111/j.1538-7836.2008.02892.x [doi]. 10.1111/j.1538-7836.2008.02892.x 18194423

[27] Eitzman DT, McCoy RD, Zheng X, Fay WP, Shen T, et al.. (1996) Bleomycin-induced pulmonary fibrosis in transgenic mice that either lack or overexpress the murine plasminogen activator inhibitor-1 gene. J Clin Invest 97: 232–237. 10.1172/JCI118396 [doi]. 10.1172/JCI118396 PMC507084 8550840

[28] Allen GB, Cloutier ME, Larrabee YC, Tetenev K, Smiley ST, et al.. (2009) Neither fibrin nor plasminogen activator inhibitor-1 deficiency protects lung function in a mouse model of acute lung injury. Am J Physiol Lung Cell Mol Physiol 296: L277–L285. 90475.2008 [pii];10.1152/ajplung.90475.2008 [doi]. 10.1152/ajplung.90475.2008 PMC2660214 19060228

[29] Hua F, Ren W, Zhu L (2011) Plasminogen activator inhibitor type-1 deficiency exaggerates LPS-induced acute lung injury through enhancing Toll-like receptor 4 signaling pathway. Blood Coagul Fibrinolysis 22: 480–486. 10.1097/MBC.0b013e328346ef56 [doi]. 10.1097/MBC.0b013e328346ef56 21577093

[30] Choi G, Schultz MJ, Levi M, van der Poll T (2006) The relationship between inflammation and the coagulation system. Swiss medical weekly : official journal of the Swiss Society of Infectious Diseases, the Swiss Society of Internal Medicine, the Swiss Society of Pneumology 136: 139–144. 10.4414/smw.2006.11059 16633958

[31] Kipnis E, Guery BtP, Tournoys A, Leroy X, Robriquet L, et al (2004) Massive alveolar thrombin activation in Pseudomonas aeruginosa-induced acute lung injury. Shock (Augusta, Ga ) 21: 444–451. 10.1097/00024382-200405000-00008 15087821

[32] Robriquet L, Collet F, Tournoys A, Prangere T, Neviere R, et al (2006) Intravenous administration of activated protein C in Pseudomonas-induced lung injury: impact on lung fluid balance and the inflammatory response. Respiratory research 7: 41. 16553944 10.1186/1465-9921-7-41 PMC1435891

[33] Dixon B, Santamaria JD, Campbell DJ (2008) A phase 1 trial of nebulised heparin in acute lung injury. Critical care (London, England) 12: R64. 10.1186/cc6894 PMC2481447 18460218

[34] Dixon B, Schultz MJ, Smith R, Fink JB, Santamaria JD, et al.. (2010) Nebulized heparin is associated with fewer days of mechanical ventilation in critically ill patients: a randomized controlled trial. Crit Care 14: R180. cc9286 [pii];10.1186/cc9286 [doi]. 10.1186/cc9286 PMC3219284 20937093

